# Nutrition knowledge, diet quality and orthorexic behaviors in Polish students–a pre-post repeated cross-sectional study

**DOI:** 10.1371/journal.pone.0287165

**Published:** 2023-06-14

**Authors:** Marta Plichta, Marzena Jezewska-Zychowicz

**Affiliations:** Department of Food Market and Consumer Research, Institute of Human Nutrition Sciences, Warsaw University of Life Sciences (SGGW-WULS), Warsaw, Poland; United Arab Emirates University College of Medicine and Health Sciences, UNITED ARAB EMIRATES

## Abstract

Nutrition knowledge acquired in college students may not only help in promoting an adequate diet, but also lead to excessive preoccupation with healthy eating, which is referred to as orthorexic behaviors. This study aimed to assess the relationship between nutrition knowledge, diet quality and orthorexic behaviors among college students of food and nutrition majors. Data were collected from a sample of 131 college students through a pre-post repeated cross-sectional study conducted from 2018 to 2021. The participants were asked to complete the ORTO-6 questionnaire, the nutrition knowledge test “GAROTA”, and the Beliefs and Eating Habits Questionnaire (KomPAN). The results indicated that students’ preoccupation with healthy eating (orthorexic behaviors score) during the study period did not change, in contrast nutrition knowledge and diet quality increased. There was no correlation between the orthorexic behaviors score and the nutrition knowledge score, both at the beginning and end of the study. At the beginning of the study, the orthorexic behaviors score correlated positively with “Pro-Healthy Diet Index” and “Diet-Quality Index”, and inversely with “Non-Healthy Diet Index”. However, at the end of the study, no significant correlations were observed between these variables. It can be concluded that nutrition knowledge determined positively the quality of the diet of students in food and nutrition majors, while did not affect the occurrence of orthorexic behaviors.

## Introduction

The tendency to eat healthily is generally regarded as a desirable behavior [1]. However, when it becomes excessive, it can pose risks to health as it leads to weight loss, malnutrition, clinically significant impairments in physical, personal, social, vocational, or academic functioning [2]. Such complications may occur when a person is excessively preoccupied with the quality and purity of foods, ritualistic styles of eating, and source and composition of foods, and avoids foods that are believed to be unhealthy [3]. Dietary restrictions accompanying the preoccupation with healthy eating usually lead to an improved diet [1], however, they can build up over time resulting in the exclusion of entire food groups, such as those containing fats, salt, sugar preservatives, food additives, and animal products [4].

Several studies assessed the prevalence of excessive preoccupation with healthy eating or alternatively used term “orthorexic behaviors” (OB) in the general population, which ranged from 2% to 6.9% [5–7]. However, the majority of studies on OB were conducted in high-risk groups, mainly college students from different majors [1,4,8–11]. College population is an especially sensitive group in terms of nutrition because at this age they begin to take responsibility for their own eating behaviors [12,13]. Among them, students studying food and nutrition are considered as a subgroup who would be at a much higher risk [9,11,14–17]. Nutrition knowledge (NK) acquired by individuals during their food and nutrition majors may result in greater preoccupation or fixation with healthy eating [18–21]. These students become aware of several diseases during their education and understand the importance of a balanced diet.

The association between nutrition knowledge and the prevalence of orthorexic behaviors has not been clearly described [22–24]. A study on sports science students revealed a positive correlation between “healthy nutrition obsession”, measured by ORTO-11, and the level of nutrition knowledge [22]. Moreover, individuals with orthorexic behaviors scored higher in the assessment of nutrition knowledge compared to those with anorexia nervosa, bulimia nervosa, compulsive overeating, night eating, and others [24]. A study examining the effect of a nine-week nutrition educational program on healthcare professionals working in a hospital [23] demonstrated that the intensity of orthorexic behaviors decreased after nutrition education among the participants [23]. Considering the lack of insight into changes occurring in the nutrition knowledge and diet quality and orthorexic behaviors, the aim of this study was to evaluate how 2.5-year of studying food and nutrition majors have affected nutrition knowledge, diet quality and prevalence of orthorexic behaviors among college students and the relationship between these variables. To the best of our knowledge, this type of study has not been carried out so far. We put forward the following hypotheses:

H1. A 2.5-year period of study in food and nutrition majors affects the increase in nutrition knowledge, diet quality and the prevalence of orthorexic behaviors;
H2. People with a higher prevalence of orthorexic behaviors are characterized by a higher level of nutrition knowledge;
H3. The diet of people with a higher prevalence of orthorexic behaviors is characterized by a higher quality.

## Materials and methods

### Study design and sample collection

Data were collected through a 2.5-year pre-post repeated cross-sectional study. The study was conducted in four universities, which were located in northern, central, and southern Poland. Students from the first year (both full-time and part-time) participated in the study. Those students were studying food and nutrition-related majors, such as: dietetics, gastronomy and hospitality, food technology as well as human nutrition and food evaluation. The selection of participants in the study was targeted, as dictated by the research findings, in which the prevalence of orthorexic behaviors were most often reported among those studying in food and nutrition-related majors. The core programs for all majors were very similar and included such courses as: food biochemistry, sensory analysis, food microbiology, human nutrition, basics of dietetics, food chemistry, food analysis, and food toxicology. The inclusion criteria were: females and males who were in the same development period, namely in the early adulthood (between 18 and 35 years old), studying food and nutrition majors, and who provided verbal or written consent to participate, while the exclusion criteria included age below 18 and above 35 years, studying majors other than food and nutrition, and lack of written or verbal consent to participate in the study. The study protocol was approved by the Ethics Committee of Faculty of Human Nutrition and Consumer Science, Warsaw University of Life Sciences (Resolution number 45/2017).

Before the main study, a pilot study was conducted in December 2017 via a paper and pen personal interview (PAPI) involving 30 students studying food and nutrition. A pilot study was performed, reflecting all the procedures of the main study, which allowed us to verify its accuracy by identifying the following issues: 1/ simplicity of understanding the questions, 2/ simplicity of navigating the questionnaire, 3/ understanding the purpose of the study, 4/ presence of privacy concerns, 5/ appropriateness of the questionnaire design, 6/ the clarity of the arrangement of the questionnaire, and 7/ time of filling out the questionnaire. The adopted procedures were confirmed to be adequate [25], therefore, the study could proceed without modifying the study design.

The study consisted of two phases. The first phase was conducted between January and March 2018 by an academic teacher during lectures and practical classes. Data for this study phase were obtained through PAPI. The participants took approximately 30 min to complete the questionnaire. A total of 689 students who were food and nutrition majors agreed to participate in this phase of the study.

**Fig 1:**
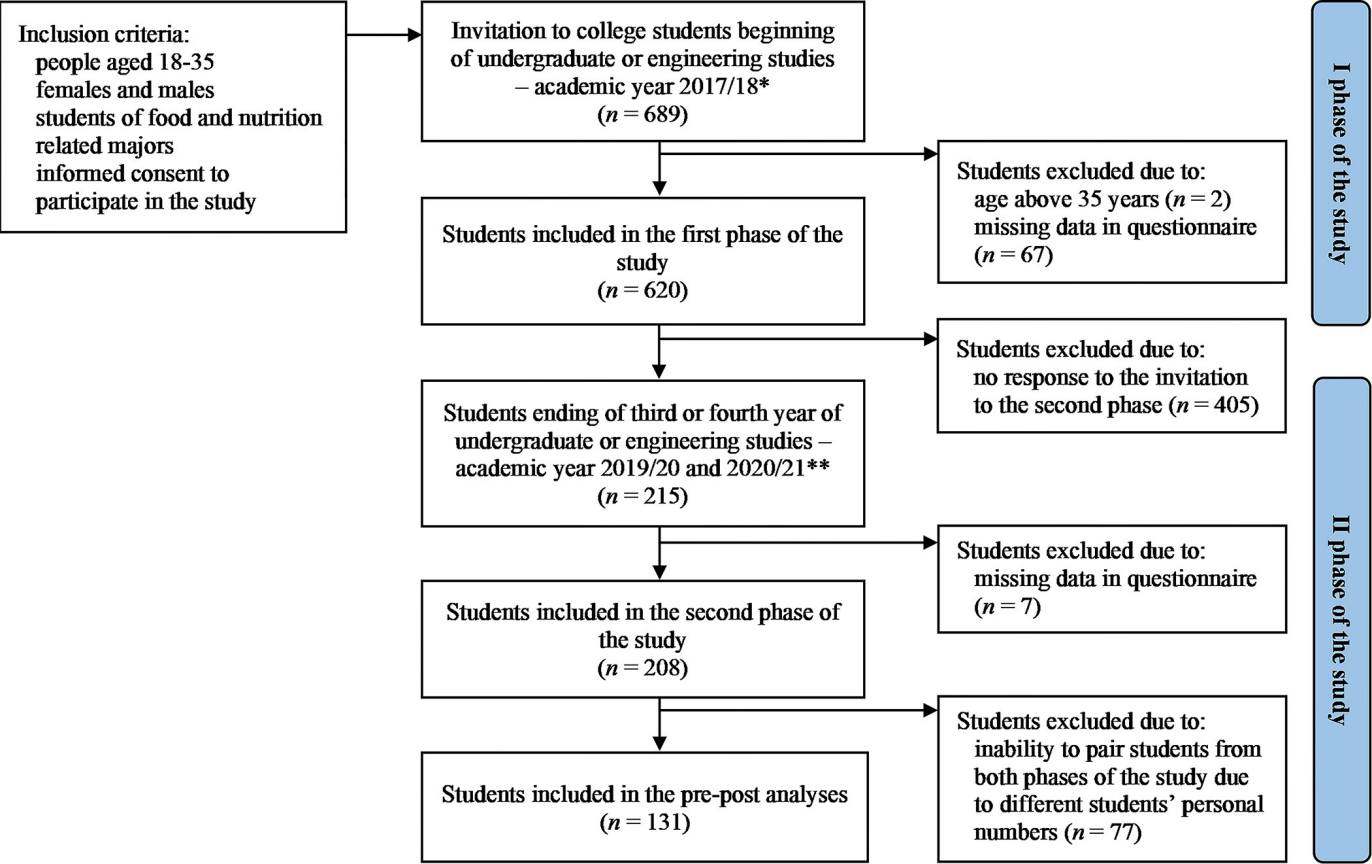
Flowchart of study design and sample collection. *the PAPI technique; **the CAWI technique; conducting the study among college students at four universities located in northern, central, and southern Poland.

Due to the COVID-19 pandemic, the second phase of the study was conducted via a computer-assisted web interview (CAWI) using a Google form between June 2020 and January 2021. The invitation to this study phase was sent to students who participated in the first phase of the study through e-mail. As the response was low, the invitation was sent five times to increase the response rates [26]. The invitation was also posted on the universities’ websites and social media platforms, such as Facebook. A total of 215 students participated in the second phase. Afterward, personal student numbers were used to look for students who had participated in both phases of the study. The final sample included in the pre-post analyses consisted of 131 students (Fig 1).

### Orthorexic behaviors

Orthorexic behaviors were assessed using the validated Polish version of the ORTO-6 questionnaire [27]. The items included in the questionnaire are as follows [28]: 1/ “In the last 3 months, did the thoughts of food worry you?”, 2/ “Are your eating choices conditioned by your worry about your health status?”, 3/ “Does the thought about food worry you for more than three hours a day?”, 4/ “Do you think that the conviction to eat only healthy food increases self-esteem?”, 5/ “Do you think that eating healthy food changes your lifestyle (frequency of eating out, friends?)”, and 6/ “Do you think that consuming healthy food may improve your appearance?”. The coding method has been reversed compared to the original ORTO-6. Responses are scored on a four-point scale as follows: 1—never, 2—sometimes, 3—often, and 4—always. The sum of scores (OB score) ranged between 6 and 24 points. Higher scores indicated a higher risk of OB. In the current sample, Cronbach’s α coefficients of the ORTO-6 for first and second phases of the study were 0.6 and 0.7, respectively.

### Nutrition knowledge

The nutrition knowledge test “GAROTA” was used to measure the level of nutrition knowledge among participants [29]. The test was developed as part of the activities of the Commission of Behavioral Determinants of Nutrition from the Polish Academy of Sciences. It was designed to evaluate the nutrition knowledge of students enrolled in food and nutrition majors and has been validated in both short and long versions [29]. The tool includes 420 questions under 14 subject categories: 1/ energy, 2/ carbohydrates, 3/ lipids, 4/ proteins, 5/ minerals, 6/ vitamins, 7/ anatomy and physiology of the digestive system, 8/ metabolism and regulation of food intake, 9/ nutritional value of food and its fortification, 10/ changes in nutritional value during food processing and storage, 11/ nutritional norms and recommendations, 12/ nutrition planning and assessment, 13/ diet-related diseases and nutrition of sick people, and 14/ other. Each category consists of 30 questions, which vary in the degree of difficulty and type as follows: 10 true/false “easy” questions; 5 true/false and 5 single-choice “moderately difficult” questions; 5 multiple-choice and 5 matching “difficult” questions [29].

In accordance with the instructions for questionnaire development, a basic version of the tool was prepared [29]. Such a version should contain 42 questions, with three questions from each subject category and each with a varying level of difficulty. In both the first and second phases of the study, single-choice questions were drawn from “moderately difficult” questions and multiple-choice questions from “difficult” questions. As recommended by the authors of “GAROTA”, different questions were asked in both phases, but they were from the same set of questions. For all types of questions, 1 point was provided for the correct answer and 0 points for wrong or no answer. The total nutrition knowledge score ranged between 0 and 42 points. Moreover, scores were summarized separately for all types of nutrition knowledge questions, obtaining a range from 0 to 14 points equally for “easy” questions, “moderately difficult” and “difficult” questions. A score of 80% or more indicated a high level of nutrition knowledge, 60–79%—moderate level, 40–59%—low level, and 39% or less—indicated an insufficient level [29]. As none of the assessed college students exhibited a high level of nutrition knowledge, this information has been omitted from the tables. In the current sample, Kuder-Richardson’s coefficients of the “GAROTA” test for first and second phases of the study were 0.5 and 0.6, respectively.

### Diet quality

The food frequency consumption was assessed using the Dietary Habits and Nutrition Beliefs Questionnaire (KomPAN) [30]. The KomPAN questionnaire assesses the habitual diet over the 12 months. The KomPAN questionnaire is composed of 24 food items, which include: 1/ food groups with a potentially beneficial influence on health (wholemeal bread, wholemeal bread rolls; buckwheat, oats, wholegrain pasta or other coarse-ground groats; milk; fermented milk beverages; fresh cheese curd products; white meat; fish; pulse-based foods; fruit; and vegetables), and 2/ food groups with a potentially negative influence on health, such as white bread and bakery products; white rice, white pasta, fine-ground groats; fast foods; fried foods; butter; lard; cheese; cold meats, smoked sausages, hot-dogs; red meat; sweets; tinned meats; sweetened carbonated or still beverages; energy drinks; and alcoholic beverages. The food items comprised all the most important food groups in the Polish diet as follows: grain products (4 items); fruit, vegetables, legumes, potatoes (4 items); dairy products (4 items); meat, fish, eggs (5 items); fats (2 items); beverages (3 items); and sweets and other products (2 items). Each participant reported habitual consumption of food items by indicating one of the six frequency categories: 1—less than once a month or never, 2–1–3 times a month, 3—once a week, 4—a few times a week, 5—once a day, and 6—a few times a day. The six original categories of food consumption frequency were converted into real numbers to obtain semiquantitative data as follows: 1–0.00, 2–0.06, 3–0.14, 4–0.50, 5–1.00, and 6–2.00 times/day [31].

Furthermore, three indexes of diet quality were calculated, following: 1/ “Pro-Healthy Diet Index” (pHDI), which is determined based on 10 food groups that can be potentially beneficial to health, 2/ “Non-Healthy Diet Index” (nHDI) is determined based on 14 food groups that can be potentially harmful to health, 3/ “Diet-Quality Index” (DQI) is determined based on 24 food groups, which include 10 groups with a potentially beneficial effect and 14 groups with a potentially harmful effect on health. In order to standardize the range of pHDI and nHDI and simplify its interpretation, the sum of the frequency of food consumption (times/day) and its expression on a scale from 0 to 100 points were calculated. A range of 0–33 points for pHDI indicated low intensity of beneficial dietary characteristics, but for nHDI it indicated low intensity of harmful dietary characteristics, 34–66 points indicated medium intensity and 67–100 indicated high intensity of these dietary characteristics. Weight scores were used in the calculations, which allowed the share of 10 components of pHDI to be the same as that of 14 components of nHDI. For DQI, the range was from -100 to 100 points. A range from -100 to -26 points indicated high intensity of nonhealthy dietary characteristics, -25 to 25 indicated low intensity of nonhealthy and prohealthy dietary characteristics, and 26 to 100 indicated high intensity of healthy dietary characteristics [30]. As none of the assessed individuals achieved high pHDI and nHDI scores, or exhibited a high intensity of nonhealthy dietary characteristics as indicated by a low DQI score, this information has been omitted from the tables. In the present sample, Cronbach’s α coefficients of the KomPAN for first and second phases of the study were 0.8 and 0.6, respectively.

### Sociodemographic characteristics

A questionnaire was used to collect participants’ sociodemographic information including gender, date of birth, place of residence (village, city with less than 100,000 citizens, city with 100,000 citizens or more), province of university, type of degree program (undergraduate or engineering, and master degree programs), year of study, college major, and weight (in kg) and height (in cm). The provinces of universities were divided into three geographic regions: north (Warmińsko-mazurskie), central (Masovian regional), and south (Lubelskie, Małopolskie). The body mass index (BMI) of the participants was calculated from their weight and height (in kg/m^2^), and based on the obtained values, the participants were categorized into three groups as per the guidelines of the World Health Organization [32]: underweight (BMI < 18.5), normal weight (BMI between 18.5 and 24.9), and overweight and obesity (BMI ≥ 25.0).

### Statistical analysis

The obtained results were presented as sample percentage (%) for categorical data or as mean and standard deviation (SD) for continuous data. The normality of the distribution of continuous variables was assessed using the normality Kolmogorov–Smirnov test, Lilliefors test, and normal probability plot. The homogeneity of variance was assessed using Levene’s test. The relationships between two independent categorical variables were determined using the Pearson chi-squared test. The differences between the mean values for two dependent samples with a normal distribution were measured using Student’s t-test for dependent variables, and for those with an abnormal distribution using Wilcoxon’s matched-pairs rank test. The differences between two dependent multicategorical variables were assessed using the marginal homogeneity test. The differences between the mean values for multiple independent samples with a normal distribution were measured using one-way analysis of variance (ANOVA) and the Bonferroni post hoc test, and for those with an abnormal distribution using Kruskal-Wallis Rank ANOVA test and by multiple comparisons of mean ranks for all samples. The association between the OB score, NK score, diet quality score, and BMI was determined at the beginning and at the end of the study using the Tau Kendall correlation coefficient. Pearson’s linear correlation was used to investigate the relationship between changes in the OB score, NK score, diet quality score, and BMI, which occurred during the study at universities. For all tests, p-values < 0.05 were considered significant. The statistical analyses were performed using STATISTICA software version 13.3 (StatSoft Inc., Tulsa, OK, USA; StatSoft, Krakow, Poland) and IBM SPSS Statistics for Windows version 28.0 (IBM Corp., Armonk, NY, USA). The G*Power software version 3.1.9.7 was used to calculate the post-hoc power analysis (Heinrich-Heine-Universität Düsseldorf, Düsseldorf, Germany). We could define the power of the analysis as the complement of the probability of making a type II error (β). It is generally accepted that the power of the test should be at least 0.80 to ensure the detection of differences and avoid a type II error [33]. Almost all the analyses achieved a power of 0.80, except for the correlations between changes in scores of pHDI and nHDI (1-β err prob = 0.78), correlations at the beginning of the study between OB score and nHDI score (0.40), and pHDI score and nHDI score (0.33), and correlations at the end of the study between pHDI and total nutrition knowledge score (0.56), nHDI score and total NK score (0.41), and DQI score and total NK score (0.74), as well as difference between mean of BMI at the beginning and end of the study (0.17).

## Results

### Sample characteristics

Table 1 presents the sociodemographic characteristics of the participants. A total of 131 college students participated in the study, of which 90.1% were females and 9.9% were males. During the study, 6.1% of students changed their major, but this did not affect the results because the changed major was also related to food and nutrition.

**Table 1:** Sociodemographic characteristics of college students from northern, central, and southern Poland in a pre-post repeated cross-sectional study (N = 131).

Variables	N	%
**Gender N (%)**		
Female	118	90.1
Male	13	9.9
**Age in years (mean ± SD)**	I phase: 20.2 ± 1.6/II phase: 22.9 ± 1.6	
**Place of residence N (%)**		
Village	53	40.5
City with less than 100,000 citizens	36	27.4
City with 100,000 citizens or more	42	32.1
**Region of university N (%)**		
North	10	7.6
Central	90	68.7
South	31	23.7
**College major N (%)**		
Dietetics	32	24.5
Food Technology	40	30.5
Gastronomy and Hospitality	24	18.3
Human Nutrition and Food Evaluation	35	26.7

N—number of participants; %—sample percentage; SD—standard deviation.

### Nutrition knowledge, orthorexic behaviors, diet quality, and BMI among college students during their study

The mean OB score of the students was 16.1 (SD = 3.3) at the beginning of the study and 15.8 (SD = 3.1) at the end. The BMI of the students increased during the study period (p = 0.044). At the end of the study, the students obtained significantly higher scores for “easy” NK questions (p < 0.001) and “moderately difficult” NK questions (p < 0.001), as well as a higher total NK score (p < 0.001). The percentage of students with insufficient and low level of NK decreased (from 31.3% to 9.9% and 61.8% to 48.9%, respectively), while the percentage with moderate level NK increased (from 6.9% to 41.2%, p < 0.001) during their study (Table 2).

**Table 2:** Pre-post comparison of nutrition knowledge, orthorexic behaviors, diet quality, and BMI in the college students from northern, central, and southern Poland (N = 131).

Variables	Study period		P-value
	Beginning of the Study	End of the Study	
**OB score (mean ± SD)** *	16.1 ± 3.3	15.8 ± 3.1	0.277
**BMI in kg/m**^**2**^ (mean ± SD)*	21.5 ± 3.2	21.8 ± 3.5	0.044
**BMI categories N (%)** **			
Underweight	16 (12.2)	9 (6.9)	0.052
Normal weight	104 (79.4)	111 (84.8)	
Overweight/obesity	11 (8.4)	11 (8.3)	
**Nutrition knowledge (mean ± SD)** *			
„Easy”questions	10.2 ± 1.9	11.1 ± 2.0	< 0.001
„Moderately difficult”questions	5.7 ± 1.9	9.1 ± 2.3	0.000
„Difficult”questions	3.0 ± 1.9	3.0 ± 1.9	0.680
Total score	18.9 ± 3.9	23.1 ± 4.9	< 0.001
**Level of nutrition knowledge N (%)** **			
Insufficient	41 (31.3)	13 (9.9)	< 0.001
Low	81 (61.8)	64 (48.9)	
Moderate	9 (6.9)	54 (41.2)	
**pHDI (mean ± SD)** *	25.8 ± 11.1	30.1 ± 12.8	< 0.001
**Level pHDI N (%)** **			
Low	101 (77.1)	79 (60.3)	0.002
Medium	30 (22.9)	52 (39.7)	
**nHDI (mean ± SD)** *	15.8 ± 8.6	12.5 ± 7.5	< 0.001
**Level of nHDI N (%)** **			
Low	128 (97.7)	130 (99.2)	0.317
Medium	3 (2.3)	1 (0.8)	
**DQI (mean ± SD)** ***	10.1 ± 14.8	17.5 ± 14.6	< 0.001
**Level of DQI N (%)** **			
Low intensity of nHDI and pHDI	114 (87.0)	91 (69.5)	< 0.001
High intensity of pHDI	17 (13.0)	40 (30.5)	

N—number of participants; %—sample percentage; SD—standard deviation; BMI—Body mass index; pHDI—Pro-Healthy Diet Index; nHDI—Non-Healthy Diet Index; DQI—Diet-Quality Index; OB—Orthorexic behaviors; * Wilcoxon’s matched-pairs rank test

** The marginal homogeneity test

*** Student’s t-test for dependent variables.

An increase in pHDI (p < 0.001) and DQI (p < 0.001) scores was observed among the students. There was a decrease in the percentage of students with a low level of pHDI from 77.1% to 60.3%, while the percentage of those with a medium level of pHDI increased from 22.9% to 39.7% (p = 0.002). Similarly, the percentage of individuals who exhibited a low intensity of nonhealthy and prohealthy dietary characteristics (DQI) decreased from 87.0% to 69.5%, while the percentage of those displaying a high intensity of healthy dietary characteristics (DQI) increased from 13.0% to 30.5% (p < 0.001). A decrease in the nHDI score was observed at the end of the study (p < 0.001). No differences were observed between students in the OB score, BMI categories, the score obtained for “difficult” NK questions, and the level of nHDI score at the beginning and the end of the study (Table 2).

### Orthorexic behaviors, diet quality, and BMI in relation to nutrition knowledge at the beginning and the end of the study

At the beginning of the study, students with insufficient NK achieved higher mean OB scores compared to those with low NK (p = 0.030). No differences in the OB score were observed between students with moderate NK and others. Students with moderate NK had a lower BMI compared to others. No differences in pHDI, nHDI, and DQI were observed between students across the level of nutrition knowledge at the beginning of the study (Table 3).

**Table 3:** Pre-post comparison of orthorexic behaviors, diet quality, and BMI among college students from northern, central, and southern Poland across the level of nutrition knowledge (N = 131).

Variables	Level of Nutrition Knowledge at the Beginning of Study			P-value	Level of Nutrition Knowledge at the End of Study			P-value
	Insufficient N = 41	Low N = 81	Moderate N = 9		Insufficient N = 13	Low N = 64	Moderate N = 54	
**OB score (mean ± SD)** *	17.2 ± 2.9 ^a^	15.6 ± 3.2 ^b^	15.6 ± 4.7 ^a,b^	0.030	16.1 ± 3.3	15.9 ± 2.9	15.5 ± 3.2	0.560
**BMI in kg/m**^**2**^ (mean ± SD)*	21.9 ± 2.0 ^a^	21.9 ± 3.5 ^a^	19.3 ± 1.1 ^b^	0.012	23.4 ± 3.5	21.7 ± 2.9	21.5 ± 4.2	0.062
**BMI categories N (%)** **								
Underweight	4 (9.8)	10 (12.4)	2 (22.2)	0.638	0 (0.0)	5 (7.8)	4 (7.4)	0.432
Normal weight	35 (85.4)	62 (76.5)	7 (77.8)		10 (76.9)	54 (84.4)	47 (87.0)	
Overweight/obesity	2 (4.8)	9 (11.1)	0 (0.0)		3 (23.1)	5 (7.8)	3 (5.6)	
**pHDI (mean ± SD)** *	26.5 ± 12.6	24.9 ± 10.1	30.2 ± 11.6	0.394	24.3 ± 14.7 ^a^	28.4 ± 13.4 ^a,b^	33.5 ± 10.8 ^b^	0.015
**Level pHDI N (%)** **								
Low	31 (75.6)	65 (80.3)	5 (55.6)	0.238	9 (69.2)	44 (68.7)	26 (48.2)	0.058
Medium	10 (24.4)	16 (19.7)	4 (44.4)		4 (30.8)	20 (31.3)	28 (51.8)	
**nHDI (mean ± SD)** *	17.3 ± 10.2	15.4 ± 7.4	12.1 ± 9.9	0.335	17.7 ± 9.2 ^a^	13.0 ± 7.3 ^a,b^	10.7 ± 6.6 ^b^	0.020
**Level of nHDI N (%)** **								
Low	39 (95.1)	80 (98.8)	9 (100)	0.398	12 (92.3)	64 (100)	54 (100)	0.010
Medium	2 (4.9)	1 (1.2)	0 (0.0)		1 (7.7)	0 (0.0)	0 (0.0)	
**DQI (mean ± SD)** ***	9.3 ± 15.6	9.6 ± 13.7	18.1 ± 19.3	0.107	6.6 ± 9.9 ^a^	15.3 ± 15.1 ^a,b^	22.7 ± 12.9 ^b^	0.003
**Level of DQI N (%)** **								
Low intensity of nHDI and pHDI	38 (92.7)	70 (86.4)	6 (66.7)	0.106	13 (100)	48 (75.0)	30 (55.6)	0.003
High intensity of pHDI	3 (7.3)	11 (13.6)	3 (33.3)		0 (0.0)	16 (25.0)	24 (44.4)	

N—number of participants; %—sample percentage; SD—standard deviation; BMI—Body mass index; pHDI—Pro-Healthy Diet Index; nHDI—Non-Healthy Diet Index; DQI—Diet-Quality Index; OB—Orthorexic behaviors

* Kruskal-Wallis Rank ANOVA test, and multiple comparisons of mean ranks for all samples

** The Pearson chi-squared test

*** The one-way analysis of variance (ANOVA), and Bonferroni test; ^a,b^ the means differ statistically significantly at p < 0.05.

At the end of the study, students with moderate NK were characterized by a higher mean pHDI score compared to those with insufficient NK (p = 0.015). On the other hand, no differences in pHDI scores were observed between students with low NK and others. Students with insufficient NK obtained a higher nHDI score compared to those with moderate NK (p = 0.020). None of these students with low and moderate NK was characterized by a medium level of nHDI compared to those with insufficient NK (p = 0.010). Students with moderate NK had a higher DQI score than those with insufficient NK (p = 0.003). More students with insufficient NK achieved low nHDI and pHDI compared to others at the end of the study (p = 0.003) (Table 3).

### Correlations between orthorexic behaviors, nutrition knowledge, diet quality, and BMI among college students at the beginning and the end of the study

The correlations observed between the OB score, BMI, total NK score, pHDI score, nHDI score, and DQI score are shown in Table 4. At the beginning of the study, a positive correlation was observed between the OB score and the pHDI score (r = 0.263), as well as the DQI score (r = 0.268). However, the OB score was inversely correlated with the nHDI score (r = -0.130). The pHDI score was correlated positively with the DQI score (r = 0.370) and inversely with the nHDI score (r = -0.133). An inverse correlation was observed between the nHDI score and the DQI score (r = -0.497). At the end of the study, the pHDI score was positively correlated with the DQI score (r = 0.683) and the total NK score (r = 0.183). On the other hand, the nHDI score was inversely correlated with the DQI score (r = -0.334) and the total NK score (r = -0.151). A positive correlation was observed between the DQI score and the total NK score (r = 0.225).

**Table 4:** Tau Kendall correlations between orthorexic behaviors, nutrition knowledge, diet quality, and BMI among college students from northern, central, and southern Poland in a pre-post repeated cross-sectional study (N = 131).

Items	OB Score	BMI	pHDI Score	nHDI Score	DQI Score	Total Nutrition Knowledge Score
**Begining of the Study**						
OB score	-	-0.023	0.263 ***	-0.130 *	0.268 ***	-0.112
BMI		-	-0.005	0.024	-0.003	-0.066
pHDI score			-	-0.133 *	0.370 ***	0.029
nHDI score				-	-0.497 ***	-0.091
DQI score					-	0.067
Total nutrition knowledge score						-
**End of the Study**						
OB score	-	-0.042	0.068	0.009	0.074	-0.031
BMI		-	-0.022	0.031	-0.052	-0.089
pHDI score			-	-0.015	0.683 ***	0.183 *
nHDI score				-	-0.334 ***	-0.151 *
DQI score					-	0.225 **
Total nutrition knowledge score						-

* p < 0.05

** p < 0.001

*** p < 0.0001

BMI—Body mass index; pHDI—Pro-Healthy Diet Index; nHDI—Non-Healthy Diet Index; DQI—Diet-Quality Index; OB—Orthorexic behaviors.

The relationships between changes in measured variables are presented in Table 5. A positive correlation was observed between the changes in the OB score and the pHDI (r = 0.323), and DQI (r = 0.293). Similarly, there was a positive correlation between changes in the pHDI and changes in the nHDI (r = 0.238), and DQI (r = 0.812) However, changes in the nHDI were inversely correlated with changes in the DQI (r = -0.373) (Table 5).

**Table 5:** Pearson’s correlations between changes in orthorexic behaviors, nutrition knowledge, diet quality, and BMI in the college students from northern, central, and southern Poland in a pre-post repeated cross-sectional study (N = 131).

Items	Change in OB Score	Change in BMI	Change in pHDI Score	Change in nHDI Score	Change in DQI Score	Change in Total Nutrition Knowledge Score
Change in OB score	-	-0.049	0.323 ***	0.025	0.293 *	-0.136
Change in BMI		-	-0.092	0.001	-0.089	-0.057
Change in pHDI score			-	0.238 *	0.812 ***	0.079
Change in nHDI score				-	-0.373 ***	0.106
Change in DQI score					-	0.013
Change in total nutrition knowledge score						-

* p < 0.05

** p < 0.001

*** p < 0.0001; BMI—Body mass index; pHDI—Pro-Healthy Diet Index; nHDI—Non-Healthy Diet Index; DQI—Diet-Quality Index; OB—Orthorexic behaviors.

## Discussion

This study aimed to assess the relationship between nutrition knowledge, diet quality and prevalence of orthorexic behaviors among college students who studying food and nutrition majors over 2.5 years. It has been already confirmed that food and nutrition students are particularly at a higher risk of developing orthorexic behaviors [9,11,16,17,34]. The changes in the prevalence of OB when the students were studying food and nutrition majors courses were also noted [35]. In the study on undergraduate dietetics students [35] an initial decrease in the prevalence of OB during the second year (p > 0.05) was observed, and then an increase appeared in the third year. The master’s study favored a linear decrease in the prevalence of OB, however, the difference was not significant. Similarly to results of our study, no significant differences in the prevalence of orthorexic behaviors were observed also in the study of Skrzypek et al. [36]. A cross-sectional study showed, however, that students studying nutrition as well as nutrition and home economics in the seventh or above semester had significantly lower OB intensity compared to students in the first and second semester [11]. Due to the fact that some students complete their education in the first-degree program, it can be expected that they may continue to exhibit increased orthorexic behaviors in the future. This may be one possible explanation for the higher prevalence of orthorexic behaviors among professional dietitians [19,37]. Moreover, dietitians may be that they are more exposed to the demands related to diet quality compared to other populations [38].

Our study did not show any correlation between the OB score and the level of nutrition knowledge, either at the beginning or at the end of the study. The lack of correlation between NK and OB, may indicate that factors other than NK may determine the prevalence of OB. This is supported by the results of other research indicating a weak association [39–41] or a lack of association between NK and OB [42,43]. Thus, further research should look for other determinants of orthorexic behaviors among dietetic and medical students, as well as among people working in public health. Hitherto, longitudinal research in a group of healthcare professionals has rarely been conducted. Nonetheless, a nine-week nutrition education was shown to result in a significant decrease in the prevalence of orthorexic behaviors among healthcare professionals (from 11.7% to 0.9%) [23]. However, this study did not measure the level of nutrition knowledge before and after the nutrition education [23].

Much research have emphasized the association between orthorexic behaviors and attitudes towards food and nutrition [44–49]. Thus, it can be assumed that these attitudes, compared to nutrition knowledge, may demonstrate stronger relationship with the prevalence of OB both before and during the food and nutrition study. Therefore, future research should assess, besides NK, the attitudes toward nutrition and health, and their changes during the study. Despite the lack of association between the total score for nutrition knowledge and orthorexic behaviors, differences in the prevalence of orthorexic behaviors were observed between groups of students with different levels of NK, but only at the beginning of the study. At the beginning of the study, the prevalence of OB was higher in those with insufficient NK compared to those with low knowledge.

As expected, studying food and nutrition majors increased the nutrition knowledge of participants. There was an increase in the total NK score, as well as in the scores obtained for easy and moderately difficult questions. In contrast, there was no change in the score obtained for questions that relate to very complex nutritional issues, the so-called difficult questions (e.g. “The requirement for which vitamins are specified in milligrams or micrograms?”, “What secretory cells are the pancreatic islets of Langerhans composed of?” or “What is the energy equivalent of sugar alcohols (polyols) used as sweeteners?”). As a result, none of the participants represented a high level of nutrition knowledge at the end of the study. This may suggest the need to consider changing the criteria for distinguishing the different levels of NK proposed by the authors of the tool. Nutrition knowledge was correlated with all indices of diet quality (pHDI, nHDI, DQI), but only at the end of the study. We suspect that the lack of such correlations at the beginning of the study period could be related to the role played by the home environment. Living together with parents is associated with the disclosure of similar eating behaviors, which is due, among others, to the availability of food, parental practices and family habits [50]. Nevertheless, in our study, we did not verify whether the students lived with their families or not, therefore we cannot explicitly indicate this as the reason. However, the change in living conditions resulting from a change in the place of residence, which often accompanies studying, may be conducive to increasing the importance of NK in conditioning the students’ diets. In our study, NK correlated positively with pHDI and DQI, while negatively with nHDI. However, similar to other research, this correlation was weak [39–41]. Students ending their study with a moderate level of NK exhibited a higher intensity of beneficial dietary characteristics (pHDI) as well as a lower intensity of harmful dietary characteristics (nHDI) compared to those with insufficient NK. These students also achieved a higher DQI score than students with insufficient NK, which indicates that they exhibited a lower intensity of nHDI. This is similar to the findings observed in the research by Kresić et al. [51]. Students with the highest NK more likely followed a diet that complied with the dietary recommendations compared to students with the lowest level of knowledge [51]. Although our results indicate a weak correlation between NK and diet quality, the diet of the students at the end of the study was characterized by a better quality (DQI) than that of the students who began the study. Obviously, not only education-related factors, among them a change in the living environment and food availability, may have favored an improvement in diet quality [52,53]. However, education seems to make a significant role in terms of nutrition knowledge and diet quality. Academic programs of food and nutrition-related majors, which were included in our study focus on teaching students the interrelationship between food and nutrition and the human body at the molecular, cellular, tissue, whole organism and population levels. The range of content is extensive and very detailed, which definitely increases nutrition knowledge considerably and helps to apply the rules of healthy eating.

Orthorexic behaviors score correlated positively with pHDI and DQI, and inversely with nHDI only at the beginning of the study period. However, the changes in OB, pHDI, and DQI scores between the beginning and the end of the study were positively correlated, what can be considered a positive trend in nutrition. Moreover, the weak correlation between the OB score and the nHDI score at the beginning of the study and the lack of this correlation at the end of the study, as well as the lack of correlation between changes in the OB score and in the nHDI score, may indicate that no risk of dietary deterioration resulting from increased orthorexic behaviors was observed. It is worth noting that the second phase of our study was conducted during the COVID-19 pandemic, which could also have affected the students’ eating attitudes and behaviors. Another study conducted during the COVID-19 pandemic showed that students who had a higher prevalence of OB consumed more legumes, nuts and seeds, dark green leafy vegetables, fruits and vegetables rich in vitamin A, milk and milk products [54]. A well-balanced and varied diet is important to provide a healthy immune system, which is especially important during the pandemic [54,55]. The fear and stress caused by the pandemic may lead to an increase in healthy eating obsession [56,57]. Nevertheless, the lack of correlation between the OB score and diet quality indices at the end of the study, and an improvement in these variables, may indicate that factors other than preoccupation with healthy eating may determine diet quality.

### Strengths and limitation

The strength of our study is its 2.5-year pre-post repeated cross-sectional design, which allows us to observe the changes that occurred during the study. Moreover, to the best of our knowledge, our study is the first to assess the effect of nutrition knowledge on the prevalence of orthorexic behaviors and evaluate diet quality among college students studying food and nutrition.

Nevertheless, the study has some limitations. The data used for the analysis were collected from students who were food and nutrition majors, so they could not be generalized to the whole student population. Additionally, the majority of participants were females (90.1%), which indicates a large gender disproportion, and thereby, makes it difficult to relate the results of the study also to males. Moreover, a self-reported weight and height were used for calculating BMI. In addition, due to the COVID-19 pandemic, it was necessary to change the study method from the CAPI to the CAWI, which contributed to a significant decrease in the response rate. Another limitation of the study resulting from a change in the survey method was the sample size. Although a large number of students participated in the first phase of the study, only a small sample was qualified for the pre-post analyses. This limitation is related to another, which is that not all analyses have achieved a power of 0.80. However, maintaining the large number of students from the first phase of the study would allow us to achieve the required power for all analyses.

## Conclusions

Our study showed an increase in nutrition knowledge and a lack of changes in the prevalence of orthorexic behaviors between the beginning and the end of the study period. The increase in NK did not correlate with changes in the OB score. This finding may indicate that an increase in NK is insufficient to change the prevalence of orthorexic behaviors. In contrast, nutrition knowledge showed positive correlations with diet quality indices such as “Pro-Healthy Diet Index” (pHDI) and “Diet-Quality Index” (DQI), and a negative correlation with “Non-Healthy Diet Index” (nHDI) at the end of the study. These results confirm the relevance of providing nutrition education to improve students’ diet quality, although increased nutrition knowledge did not show an association with orthorexic behaviors. At the beginning of the study, orthorexic behaviors score showed a positive correlation with pHDI and DQI, and a negative correlation with nHDI. These findings, along with a low OB score, imply that preoccupation with healthy eating has no negative consequences characteristic of orthorexia. The lack of association between orthorexic behaviors and diet quality indices at the end of the study, as well as the lack of associations between changes in the OB score and changes in nHDI over the course of the study, may indicate that other factors as such, and not just the major may contribute to an improvement in diet quality indices.

Similarly, the lack of relationships between orthorexic behaviors, and NK as well as diet quality indices at the end of the study may indicate that other factors related to food and nutrition study as such, and not just the major, may cause an improvement in the quality of students’ diet. Future research should take these factors into account, including changes in the living environment and availability of food.

## Supporting information

S1 Database (XLSX)
